# Good manufacturing practice-grade generation of CD19 and CD123-specific CAR-T cells using piggyBac transposon and allogeneic feeder cells in patients diagnosed with B-cell non-Hodgkin lymphoma and acute myeloid leukemia

**DOI:** 10.3389/fimmu.2024.1415328

**Published:** 2024-08-13

**Authors:** Martin Mucha, Martin Štach, Iva Kaštánková, Jana Rychlá, Jan Vydra, Petr Lesný, Pavel Otáhal

**Affiliations:** ^1^ Institute of Hematology and Blood Transfusion, Prague, Czechia; ^2^ Faculty of Science, Charles University, Prague, Czechia

**Keywords:** CAR-T cells, leukemia, lymphoma, electroporation, PiggyBac PB transposon

## Abstract

**Background:**

The non-viral production of CAR-T cells through electroporation of transposon DNA plasmids is an alternative approach to lentiviral/retroviral methods. This method is particularly suitable for early-phase clinical trials involving novel types of CAR-T cells. The primary disadvantage of non-viral methods is the lower production efficiency compared to viral-based methods, which becomes a limiting factor for CAR-T production, especially in chemotherapy-pretreated lymphopenic patients.

**Methods:**

We describe a good manufacturing practice (GMP)-compliant protocol for producing CD19 and CD123-specific CAR-T cells based on the electroporation of transposon vectors. The lymphocytes were purified from the blood of patients undergoing chemotherapy for B-NHL or AML and were electroporated with piggyBac transposon encoding CAR19 or CAR123, respectively. Electroporated cells were then polyclonally activated by anti-CD3/CD28 antibodies and a combination of cytokines (IL-4, IL-7, IL-21). The expansion was carried out in the presence of irradiated allogeneic blood-derived mononuclear cells (i.e., the feeder) for up to 21 days.

**Results:**

Expansion in the presence of the feeder enhanced CAR-T production yield (4.5-fold in CAR19 and 9.3-fold in CAR123). Detailed flow-cytometric analysis revealed the persistence of early-memory CAR-T cells and a low vector-copy number after production in the presence of the feeder, with no negative impact on the cytotoxicity of feeder-produced CAR19 and CAR123 T cells. Furthermore, large-scale manufacturing of CAR19 carried out under GMP conditions using PBMCs obtained from B-NHL patients (starting number=200x10e6 cells) enabled the production of >50x10e6 CAR19 in 7 out of 8 cases in the presence of the feeder while only in 2 out of 8 cases without the feeder.

**Conclusions:**

The described approach enables GMP-compatible production of sufficient numbers of CAR19 and CAR123 T cells for clinical application and provides the basis for non-viral manufacturing of novel experimental CAR-T cells that can be tested in early-phase clinical trials. This manufacturing approach can complement and advance novel experimental immunotherapeutic strategies against human hematologic malignancies.

## Introduction

The efficiency of CAR-T cell production hinges on the quality of the source material obtained from patients. Intensive chemotherapy-induced lymphopenia escalates the likelihood of CAR-T manufacturing failure and diminishes the therapy’s effectiveness (1). These factors are critical, particularly for CAR-T production based on less efficient non-viral approaches that employ electroporation of transposable DNA elements like Sleeping Beauty or PiggyBac transposons (2–4). These alternative approaches offer rapid and cost-effective manufacturing, making them suitable for early-phase clinical trials involving novel genetically engineered tumor-reactive T cells. However, effectively utilizing the non-virally-produced CAR-T necessitates novel and improved production processes.

The viral vectors face significant hurdles in their clinical application, such as large-scale vector production and complex biosafety characterization, which impact the availability of clinical-grade vector production. The standard clinical-grade manufacturing of novel types of CAR-T utilizing viral vectors is thus primarily limited by the complexity of the production of viral vectors. In contrast, transposons provide significant advantages compared to viral vectors, such as decreased production costs, increased biosafety, and low immunogenicity. Furthermore, both lentiviruses (LV) and retroviruses (RV) have lower integration capacity, and they often cannot fit more than 8–9 kb (5) compared to transposons, which additionally limits LV/RVs use for complex multi-gene modifications.

However, the use of transposons for large-scale CAR-T manufacturing faces several critical manufacturing issues. The delivery of the transposon vectors by electroporation is far more toxic to T cells than transduction with LV/RVs and requires a specific device - an electroporator. Electroporation cannot be easily performed in a large volume, i.e., this method significantly reduces the starting numbers of T cells in the manufacturing process and, therefore, yields a much lower number of CAR-T cells compared to LV/RVs (6). This low-efficiency production, unfortunately, becomes a critical factor during CAR-T manufacturing for heavily pretreated patients involved in clinical trials who have undergone intensive chemotherapies and are commonly lymphopenic.

In this study, we aimed to improve the non-viral CAR-T production based on the electroporation of transposon vectors (7), and we present a GMP-compliant production process of CD19-specific (CAR19) and CD123-specific (CAR123) CAR-T cells utilizing lethally irradiated allogeneic PBMCs obtained from healthy blood donors (referred to as the “feeder”). Our results demonstrate that the electroporation approach is highly efficient when producing CAR-T cells from T lymphocytes derived from healthy donors compared to patient-derived T lymphocytes. Previous chemotherapies induced lymphopenia in the blood of the B-NHL and AML patients (B-NHL, n=8, median 0.63x10e6 CD3+/µl, AML, n=10, median 1.3x10e6 CD3+/µl) and reduced the percentage of Tscm-like lymphocytes (CD45RA+CD62L+) which critically reduced the outcome of CAR-T manufacturing. Adding the feeder improved the expansion process and increased the CAR-T yield (4.5-fold in CAR19, n=8, p=0.007 and 9.3-fold in CAR123, n=10, p=0.012). The majority of generated CAR-T cells maintained an early memory (CD45RA+CD62L+/-) phenotype (87% of CD4+CAR19, 93% of CD8+CAR19 cells, n=8, 64% of CD4+CAR123 cells, 80% of CD8+CAR123 cells, n=10). Notably, the large-scale manufacturing of CAR19 carried out under GMP conditions using PBMCs (200x10e6 starting number) obtained from B-NHL patients enabled production of >50x10e6 CAR19 in 7 out of 8 cases in the presence of the feeder while only in 2 out of 8 cases without the feeder and this increased production efficiency made it possible to include these patients into a clinical trial (NCT05054257).

In summary, non-viral/electroporation methods for CAR-T production exhibit low production efficiency in lymphopenic patients undergoing chemotherapy compared to healthy donors. This negative factor can be partially mitigated by adding the feeder, which enhances CAR-T production output, reduces manufacturing failure, and is GMP-compatible. The observed improved expansion of CAR19 and CAR123 T cells in the presence of the feeder widens the range of potential clinical applications for this non-viral manufacturing technique, particularly for novel experimental CAR-T cell products in heavily pretreated patients.

## Results

**Figure 1:**
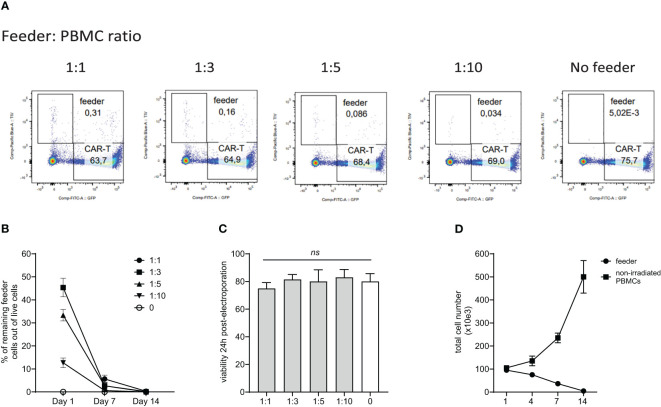
Persistence of the feeder cells during *in vitro* expansion of CAR-T cells. PBMCs from healthy donors were electroporated with CAR19 and GFP expressing transposons and mixed with decreasing feeder cells labeled with a blue fluorescent dye (TagItViolet – TIV). The percentage of feeder cells was determined on days 1, 7, and 14 - the representative dot-plot in **(A)** shows the remaining number of feeder cells at day 14 after electroporation, and the kinetics of the feeder persistence is shown in **(B)** (n=3, +/- SD). Next, the effect of the feeder on the viability of electroporated cells was evaluated - the results shown in **(C)** demonstrate that the viability post-electroporation was not significantly influenced by the presence of the feeder (n=3, ns, not significant, +/- SD, one-way ANOVA test). **(D)** Furthermore, we determined the ability of the feeder cell to proliferate *in vitro*. Feeder cells or control non-irradiated PBMCs from the same donors were polyclonally activated with anti-CD3/CD28 antibodies and expanded *in vitro* (n=3, +/- SD). The presented data confirm the inability of feeder cells to proliferate and persist.

Although the allogeneic feeder cells are lethally irradiated by 30Gy, we confirmed their inability to expand and persist during CAR-T production. The feeder cells were initially labeled with blue fluorescent TIV (Tag-It-Violet) dye. Subsequently, PBMCs were electroporated with a transposon plasmid encoding CAR19 and a second transposon plasmid encoding GFP to fluorescently label generated CAR-T cells. The TIV-labeled feeder was mixed with electroporated cells at ratios of 1:1, 1:3, 1:5, and 1:10 or not added. Flow cytometry analysis at days 1, 7, and 14 post-electroporation quantified the remaining live feeder cells. Results showed that feeder cells do not proliferate and are effectively eliminated, even at the highest 1:1 ratio during cultivation with CAR-T cells (**Figures 1A, B, D**). Additionally, the viability of electroporated cells one day post-electroporation was similar regardless of the feeder being added or not added (**Figure 1C**), suggesting additional mechanisms of the feeder’s effects.

**Figure 2:**
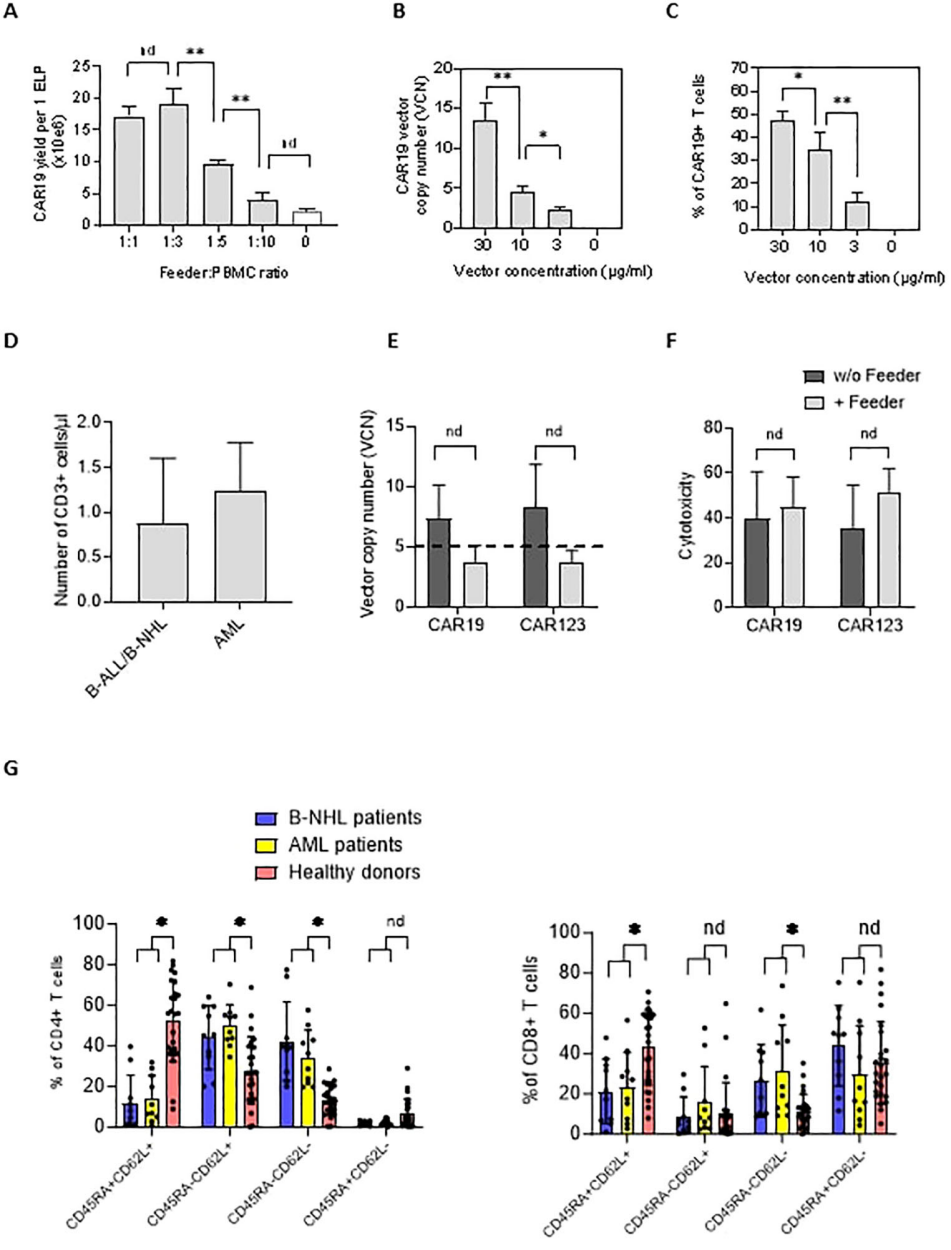
The effects of the feeder on the quality of the produced CAR19 and CAR123 T cells. **(A)** The PBMCs from B-NHL patients (n=3) were electroporated with CAR19 transposon and mixed with decreasing amounts of the feeder. The number of CAR+ T cells was determined after 14 days of expansion as a yield per one ELP. The optimal adequate amount of the feeder improving the CAR-T production was estimated to be at a 1:3 feeder: PBMCs ratio. **(B, C)** The concentration of the transposon DNA during electroporation influences the vector copy number (VCN) and the percentage of transfected T cells. The optimal concentration of the transposon vector to meet the VCN limits=5 and to enable effective transfection was determined to be 10 µg/ml (n=3). **(D)** The graph presents the median number and range of CD3+ T lymphocytes in blood samples used for low-scale production of CAR-T cells obtained from B-NHL and AML patients (n=10). Both groups of patients were lymphopenic as a result of previous chemotherapies. **(E)** To evaluate the effects of the feeder on the transposition efficiency, we measured the vector copy number (VCN) per one CAR19+ and CAR123+ T cell in the presence or absence of the feeder (vector concentration =10 µg/ml, PBMCs were obtained from B-NHL and AML patients (n=4)). The differences in VCN were insignificant due to the high variability of the VCN in CAR-T expanded without the feeder. However, all products expanded in the presence of the feeder had acceptable VCN (≤5). **(F)** The biological activity of produced CAR-T in the presence or absence of the feeder was determined by cytotoxic assay against RAMOS cells (CAR19) or THP-1 cells (CAR123) at 1:1 effector: target ratio after 24 hours of co-culture - no significant differences in the cytotoxicity between feeder/no-feeder produced CAR19, and CAR123 T cells were observed (n=4, nd = no difference, unpaired t test). **(G)** The T cell memory phenotype was determined to evaluate the effects of chemotherapies on the quality of T cells by staining for antigens CD45RA and CD62L on CD4+ or CD8+ T cells. Patient-derived samples contained significantly fewer CD45RA+CD62L+ T cells and significantly more T cells having more differentiated phenotype CD45RA-CD62L- in both CD4+ and CD8+ subsets, reflecting the patients' conditions. B-NHL n=8, AML n=10, **P < 0.01, *P < 0.05, nd = no difference, +/- SD, unpaired t test.

Next, we aimed to identify the feeder’s minimal amount leading to improved CAR-T expansion in chemotherapy-pretreated patients (and who were indicated for tisa-cel therapy). Isolated PBMCs were electroporated with CAR19 transposon and mixed with increasing amounts of feeder. Cells were then expanded in 24-well G-Rex plates in a low-scale protocol for 14 days; our results suggested that the CAR19 expansion correlated with the increasing number of feeder cells per well, peaking at a feeder: PBMC ratio of 1:3 (**Figure 2A**).

The genotoxicity of the transposition event significantly impacts the quality of produced CAR-T cells. This parameter can be indirectly assessed by quantifying the vector copy number (VCN) per CAR-T cell. Electroporation can be optimized to control the VCN by titrating the concentration of the DNA vector during electroporation (**Figure 2B**). However, lowering the DNA concentration reduces the percentage of T cells expressing CAR (**Figure 2C**). Therefore, efficient T cell expansion is a critical parameter for providing an effective CAR-T yield. VCN determined by ddPCR method in CD19 and CD123 CAR-T cells (**Figure 2E**) showed that both CD19 and CD123 CAR-T cells produced without the feeder had significantly higher VCN than cells produced in the presence of the feeder (CAR19: median 7.3 vs. 3.8, n=4; CAR123: median 8.3 vs. 3.7, n=4). Although these values were not significant (paired t test), all feeder-produced CAR19 and CAR123 had VCN within approved limit (≤5) which is considered a critical parameter in the quality control tests of the CAR-T. Based on these findings, the optimal concentration of transposon DNA in the electroporation solution enabling effective transfection and sufficiently low VCN was determined to be 10 µg/ml. To further evaluate the quality of the produced CAR-T cells, their biological activity was determined by a cytotoxic assay—both CAR19 and CAR123 T cells produced in the presence or absence of the feeder (**Figure 2F**) effectively killed target cells. In further experiments we used PBMCs obtained from B-NHL and AML patients – all patients received intensive chemotherapies in less than two months before providing the blood samples and both groups were lymphopenic (B-NHL, n=8, median 0.63x10e6 CD3+/µl; AML, n=10, median 1.3x10e6 CD3+/µl, **Figure 2D**). We determined the T-cell memory phenotype in these samples - significantly less stem-cell memory (Tscm) and significantly more effector memory (Tem) CD4+ and CD8+ T cells were detected (**Figure 2G**) in comparison to samples obtained from healthy donors.

### Improved protocol for the expansion of CD19-specific and CD123-specific CAR-T cells

**Figure 3:**
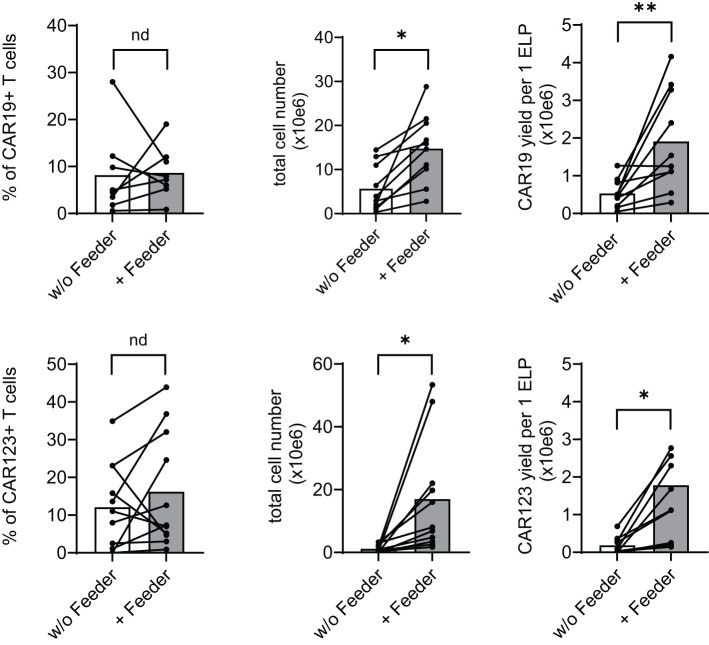
Feeder enhances the efficacy of CD19 and CD123 CAR T cell production. PBMCs obtained from B-NHL and AML patients were electroporated with CD19 CAR construct (B-NHL, top panels, N=8) and CD123 CAR construct (AML, lower panels, N=10). Cells were then polyclonally activated with TransAct and expanded in the presence or absence of the feeder for 14 days using a low-scale expansion protocol. The expansion outcome is presented as a percentage of CAR+ T cells, the total live cell numbers, and CAR-T yield per one electroporation. Adding the feeder improved total cell expansion, increasing the production yield of both CAR19 and CAR123 T cells. **P < 0.01, *P < 0.05, nd, no difference, paired t test.

The effects of the feeder on the production of CAR19 and CAR123 T cells were evaluated using a low-scale/14-day production process utilizing 24-well G-rex plates. At the end of the CAR-T expansion, we determined main parameters reflecting the efficiency of production, such as the percentage of CAR+ T cells, total number of living cells, and the yield of CAR-T per one electroporation of 1x10e7 PBMCs (**Figure 3**). In the CAR19 expansion model, a significant increase in CAR19 yield (4.5-fold) and total cell number (3.3-fold) in the presence vs. absence of the feeder was observed, while no differences in the percentage of transfected T cells were detected (**Figure 3** upper panel). In the case of CAR123, similar effects were also observed: 9.3-fold increase of the CAR123 yield and 15.4-fold increase of total cell numbers in the presence vs. absence of the feeder while minimal effects of the feeder on the percentage of transfected cells were observed (**Figure 3** lower panel).

In summary, adding the feeder increases total T-cell expansion and reduces the manufacturing failure rate. Production in the presence of the feeder has no significant negative impacts on the biological activity or the vector copy number.

### Large-scale production of CAR T cells

**Figure 4:**
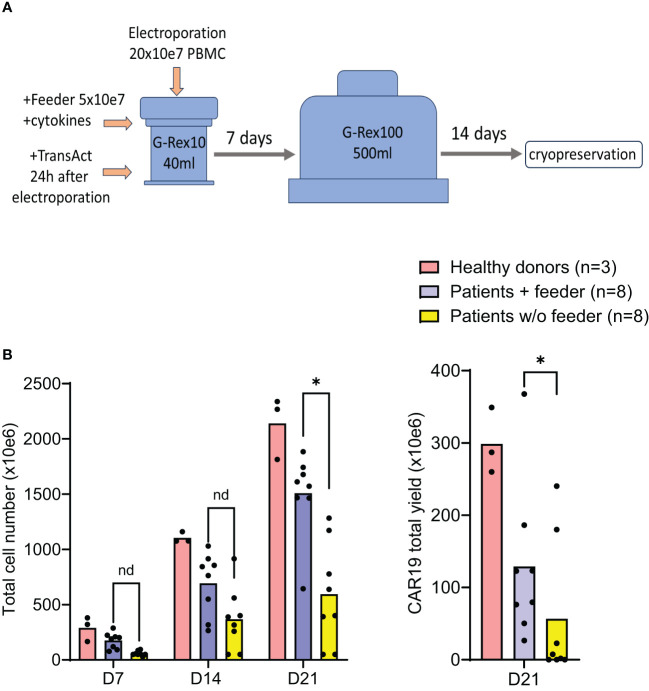
Good Manufacturing Practice-grade large-scale production of CAR19 T cells. The production efficacy of CAR19 T cells was validated by a large-scale production protocol under GMP settings. The scheme of the expansion process is shown in **(A)**. Patient-derived PBMCs (n=8) and healthy donors PBMCs (n=3) were transfected with CAR19 transposon (200 million PBMCs electroporated in 20 reactions) and expanded in G-Rex bottles in the presence of cytokines preserving the stem-cell memory phenotype (IL-4, IL-7, IL-21) for up to 21 days. The outcome is presented as a CAR19 total yield [**(A)**, right panel] and the total cell number of viable cells [**(B)** left panel]. *P < 0.05, nd, no difference, unpaired t test.

The effects of the feeder on CAR-T production were further evaluated in a large-scale GMP-certified protocol (**Figure 4A**) in patients with B-NHL involved in a clinical trial (NCT05054257). In this process, a fixed amount of the feeder cells (50x10e6) is added to G-Rex10 bottle and then is mixed with CD19 CAR transposon-electroporated PBMCs. The PBMCs were isolated from a fixed volume of peripheral blood obtained from heavily chemotherapy-pretreated patients suffering from severe T-cell lymphopenia, and based on the obtained amount of PBMCs, the feeder: PBMCs ratio in this large-scale expansion process was within the range of 1:3–1:4, i.e., 150–200 x10e6 PBMCs were electroporated. The following day, T cells were polyclonally activated and further expanded in the presence of cytokines IL-4, IL-7, and IL-21 to preserve the stem-cell memory phenotype of expanding CAR19 T cells. After seven days, the expanding cells were transferred to G-Rex100 bottles and further cultivated until Day 21, when the cells were harvested and cryopreserved. To obtain such high numbers of PBMCs these B-NHL patients underwent leukapheresis as a part of approved clinical trial with CD19 CAR-T cells and all of them had relapsed/refractory B-cell acute leukemia or B-cell lymphoma with more than 3 lines of therapies.

**Figure 5:**
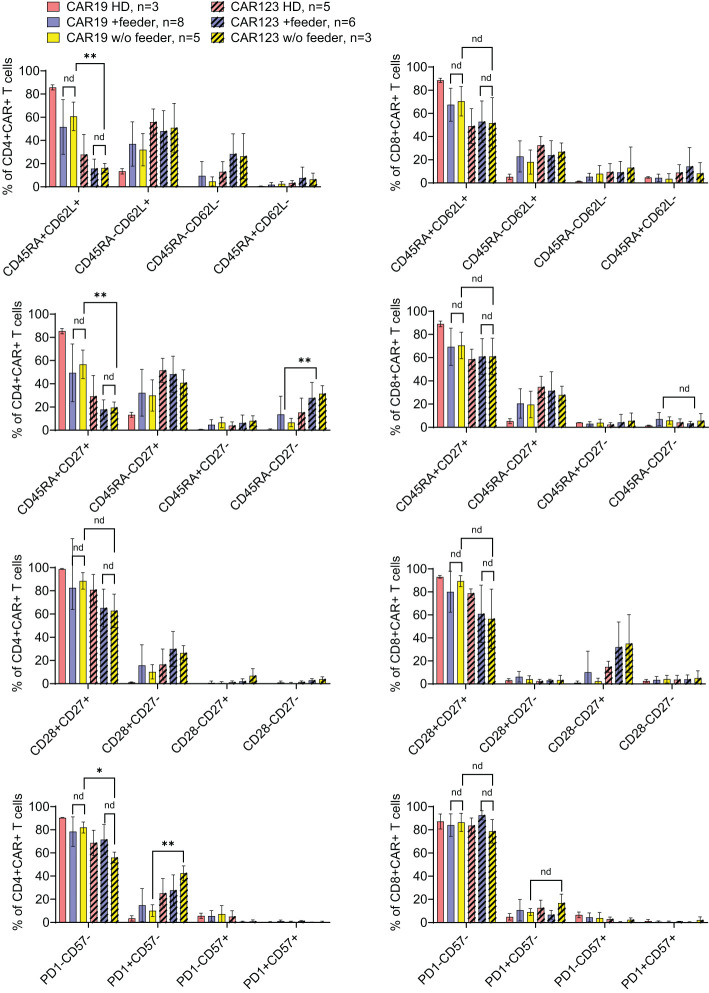
The effects of the feeder on the immunophenotype of the produced CAR T cells. CAR19, generated by the large-scale production protocol, and CAR123, generated by the low-scale production protocol, were analyzed by flow cytometry to identify the effects of the feeder on their immunophenotype. PBMCs obtained from healthy donors (HD) were used as control samples. The quantification of individual memory subsets among CAR+ T cells is presented by staining for antigens CD45RA and CD62L, CD27, CD28, PD-1, and CD57 among CD4+ or CD8+ CAR+ T cells. No significant differences in the immunophenotypes were identified between feeder/no-feeder-produced CAR19 and CAR123 T cells. However, CD4+ CAR123 T cells displayed more differentiated/exhausted phenotype in comparison to CD4+ CAR19 T cells (patient-derived/+ feeder groups). **P < 0.01, *P < 0.05, nd, no difference, unpaired t test.

The outcomes of CAR19 T cell production in these patients (n=8) are presented (**Figure 4B**). These results indicate that the addition of the feeder enables the production of >50x10e6 CAR19 in 7 out of 8 cases in the presence of the feeder while only in 2 out of 8 cases without the feeder. The effective CAR19 expansion without the feeder in these two patients was associated with the presence of high numbers of CD19+ ALL blasts in the blood, which hypothetically might have activated CAR19 during *in vitro* expansion, and no effects of the feeder were observed in these two cases. The remaining patients had minimal numbers of CD19+ B cells in the blood due to previous therapy with anti-CD20 antibody rituximab. The kinetics of T cell expansion (**Figure 4B**) suggest that the main effect of the feeder is based on the enhancement of total cell expansion rather than primarily increasing the percentage of transfected cells. As previously demonstrated, the combination of cytokines IL-4, IL-7, and IL-21 facilitates the persistence of stem-cell memory CAR-T cells during prolonged *in vitro* expansion (8). A similar analysis was performed to assess the effects of the feeder on the CD19 CAR-T differentiation pattern. Analogically, we analyzed the CAR123 T cells produced by the low-scale method, and the results are presented altogether (**Figure 5**). The CAR19 and CAR123 produced from healthy donor’s PBMCs were expanded without adding the feeder. By multicolor flow cytometry, we identified the expression of antigens CD3, CD4, CD8, CD62L, CD45RA, CD27, CD28, PD1, and CD57 to quantify T cell memory subtypes and their exhaustion patterns. No significant differences in CAR-T immunophenotypes after expansion in the presence or absence of the feeder were observed. The majority of CAR19 displayed a less-differentiated (CD45RA+CD27+CD28+PD1-) memory phenotype. However, CAR123 T cells showed more differentiated memory patterns than CAR19 cells, mainly in the CD4+ subtype (**Figure 5**). A possible explanation is that CD19 antigen is expressed by normal B cells and CD123 antigen is expressed by normal basophils – the recognition of these natural target cells by.

CAR-T during the expansion might influence the differentiation pattern (both CAR19 and CAR123 have identical 4–1BB-zeta signaling domains).

In conclusion, the large-scale expansion of CAR19 T cells in the presence of the feeder results in a more effective production process and generates cells with an optimal immunophenotype. This large-scale expansion was carried out under GMP conditions, and some of the produced CAR19 were later used in the ongoing clinical trial (NCT05054257).

## Materials and methods

### Cell source, DNA vectors, feeder production

Peripheral blood mononuclear cells (PBMCs) were isolated from buffy coats obtained from blood donors or from blood samples obtained from AML and B-ALL/B-NHL patients. All human materials used were approved by institutional review boards, and donors and patients signed informed consent for the use of their biological materials. The THP-1 and RAMOS cell lines were acquired from DFMZ, DE. The CAR19 transposon was described previously (9), the CAR123 was created by gene-synthesis using the published sequence as a source (10). The CAR sequences were assembled into parental piggyBac (PB) transposon vectors containing the UBC promoter using standard restriction endonuclease-based cloning techniques. The transposase-expressing vector contained the hyperactive piggyBac transposase driven by the CMV promoter (11, 12). Plasmids were purified using EndoFree kits (Qiagen, Germany). To produce the feeder, buffy coats were lethally irradiated (30 Gy) and processed by Ficoll gradient centrifugation. Isolated PBMCs from five healthy blood donors were mixed and cryopreserved for further use. All cells were grown in CellGenix GMP DC Media supplemented with 10% fetal calf serum and penicillin (100 I.U./mL), streptomycin 100 (μg/mL) antibiotics; the GMP-grade cultivation was without antibiotics.

### Production of CAR T cells

PBMCs were transfected with transposon vectors using the Neon electroporator (Thermo Fisher Scientific, USA), following previously established procedures (9). Briefly, 1x10e7 cells were resuspended in 100 μl buffer T containing 1 μg of CAR vector DNA and 1 μg of transposase-expressing vector DNA, then electroporated (20 reactions) using a 1x 20ms/2300V pulse setting per reaction. Subsequently, cells were cultured in G-Rex 10 bottles (Wilson Wolf, USA) in media supplemented with cytokines IL-4 (20 ng/ml), IL-7 (10 ng/ml), and IL-21 (40 ng/ml) in the presence or absence of the feeder (50 mil per G-Rex 10). The next day, cells were activated with TransAct (Miltenyi Biotec, Germany). At day 7, cells were transferred to G-Rex 100 bottles (Wilson Wolf, USA) and continually supplemented with complete media. Small-scale production involved one electroporation reaction of PBMCs obtained from approximately 5–10 ml of blood, followed by cultivation in G-Rex 24 plates (Wilson Wolf, USA) in the presence (5x10e6 cells per well) or, absence of the feeder.

### Antibodies and FACS

CAR-T cells were detected with FITC-labeled goat anti-mouse Ab (CAR19) or FITC-labeled anti-FLAG Ab (CAR123). The antigens CD4, CD8, CD45RA, CD62L, CD27, CD28, CD57, and PD1 were used to identify T cell differentiation patterns. The fluorescently labelled antibodies are listed in **Table 1**. FACS samples were analyzed with a BD Fortessa cytometer, and FlowJo software was used to process FACS data. Statistical analysis was performed using GraphPad Prism software with indicated tests.

**Table 1:** Antibody panel.

Antigen	Fluorochrome	Clone	Manufacturer	Cat. No.
CD3	BV786	UCHT1	BD	565491
CD62L	BV650	DREG-56	BD	563808
CD27	BV480	M-T271	BD	746296
CD4	Pacific Blue	RPA-T4	BD	558116
CD45RA	BUV737	HI100	BD	612846
CD28	PECy7	CD28.2	BioLegend	302926
CD14	Alexa Fluor 594	HCD14	BioLegend	325630
PD-1	PE	EH12.2H7	BioLegend	329906
CD57	APC-Vio770	REA769	Miltenyi Biotec	130–111-813
CD8	Alexa Fluor 700	MEM-31	Exbio	A7–207-T100
Anti-DYKDDDDK Tag	Alexa Fluor 488	L5	BioLegend	637318
F(ab’)2 Fragment Goat Anti-Mouse IgG (H+L)	Alexa Fluor 488		Jackson IR	115–546-003
LIVE/DEAD Fixable Blue Dead Cell Stain			Invitrogen	L34962

### *In vitro* assays

Cytotoxic tests of CAR-T cells were performed against AML cell line THP-1 (CAR123) or B-cell line RAMOS (CAR19). Target cells were labeled with CFSE dye at a concentration of 5 μM for 5 min at 37°C (Thermo Fisher, USA) and cultivated with CAR-T cells at a 1:1 effector: target ratio for 24 hours in 1 ml media in 24-w plate. The percentage of dead/live target cells was determined by FACS using DAPI live dye. The cell numbers were determined by using a common counting chamber.

### Vector copy number

A droplet digital PCR (ddPCR)-based approach was developed to measure the vector copy number (VCN) of both CAR19 and CAR123 transposons. Genomic DNA was isolated with QIAamp DNA Mini Kit (Qiagen, Germany). Duplex PCR reactions contained ddPCR Supermix for Probes (no dUTP) (cat. n. 186–3024, Bio-Rad Laboratories, USA), 900 nM of each primer pair, 250 nM of each FAM- and HEX-labeled probe, and 40 ng of genomic DNA. ddPCR equipment from Bio-Rad Laboratories (USA) was used in all tests. The reaction mix was split into around 20,000 droplets using a QX200 droplet. The PCR was performed on a C1000 Touch thermal cycler using the following amplification conditions: 10 min at 95°C, 45 cycles of 30s at 94°C and 60s at 54°C, and ending with 10 min at 98°C for droplet stabilization and cooling to 4°C. Droplets were analyzed by QX200 droplet reader based on their fluorescence amplitude into positive or negative. Data were processed with Quanta-Soft Analysis Pro software. The vector copy number (VCN) was determined as the ratio of (CAR copies/albumin copies) x2/% of CAR+ T cells in the sample.

## Discussion

In this study, we present a Good Manufacturing Practice-grade method of production of CD19 and CD123-specific CAR-T cells. Non-viral CAR-T production is facing low production efficiency - here, we show that the addition of lethally irradiated allogeneic mixed PBMCs effectively increased the production yield of both CD19 CAR-T and CD123 CAR-T in patients with low T-cell counts/quality due to previous chemotherapies and reduced the manufacturing failure rate. Importantly, no negative effects of the feeder, such as alterations in CAR-T memory phenotype, an increase in vector copy number, or reduction in CAR-T cytotoxicity, were observed. The feeder-based protocol was implemented for the production of GMP-grade therapeutic CAR19 which are currently used in a clinical trial NCT05054257.

Electroporation, a key step in the discussed CAR-T cell manufacturing process, is inherently damaging to T cells (13). Reducing this toxicity is crucial for the successful development of an efficient manufacturing process. When cells are exposed to an electric field in the presence of DNA, a DNA-membrane complex is formed on the membrane facing the cathode. This complex then enters the cells through endocytosis or macropinocytosis (14–16). The presence of DNA in the cytoplasm mitigates viral infection and triggers cell defensive pathways by activating cytosolic DNA sensors (17). These sensors, a subgroup of pattern recognition receptors (PRRs), not only induce an inflammatory immune response in damaged cells but may also lead to cell death, primarily through apoptosis (13). These effects are cell type-specific and dependent on the concentration of DNA. In addition to the mechanisms triggered by PRRs, the physical parameters of electroporation, such as the strong electric field, can cause membrane damage, resulting in electrolyte imbalance, influx of water, osmotic swelling of the cells, and, consequently, cell death by necrosis (18). The physical parameters are defined by the electroporator device’s design, such as the electroporation chamber’s width. Several instruments, such as Amaxa^®^Nucleofector^®^, MaxCyte^®^, Neon™ transfection system, and Xenon™ electroporation system, are currently available and are used to produce therapeutical CAR-T in various clinical trials. After testing these instruments, we observed that Neon can effectively electroporate non-activated T cells at low DNA concentration (10 μg/ml), resulting in a vector copy number less than 5. We have also tested the electroporation of pre-activated T cells using all of these instruments. However, the outcome was not superior to the presented protocol due to the high toxicity of this type of electroporation, which additionally required a much higher concentration of the plasmids. For these reasons, the Neon device was selected for GMP-grade CAR-T cell production.

The importance of the microenvironment is essential for ELP-based production. For example, CAR-T expansion might be supported by antigenic stimulation via CD19-positive B cells (in the case of CAR19) and CD123-positive basophils (in the cease of CAR123) which are physiologically present in the feeder and also by polyclonal T-cell stimulation by HLA-mismatch between the feeder and electroporated PBMCs. In the past, various engineered cell lines have been developed as feeders - however, the usage of such cell lines poses challenges for Good Manufacturing Practice (GMP) production. For instance, Nakamura et al. compared autologous PBMCs with a modified K562 cell line expressing costimulatory molecules CD80, CD86, CD83, and 4–1BB ligand (19). Both types of feeder cells were highly effective in supporting CD19 CAR-T expansion. Similarly, Numbenjapon et al. described artificial antigen-presenting cells (APCs) derived from K562 cells expressing CD19 antigen and two T-cell costimulatory molecules (4–1BB ligand and major histocompatibility class I–related chains A) (20). The use of this APCs led to enhanced expansion of CD19 CAR-T cells during non-viral/electroporation-based production. Morita et al. used irradiated activated autologous T cells to enhance the production of piggyBac-generated CD19-specific CAR-T cells, resulting in an increased percentage of CAR+ T cells and improved *in vitro* expansion (21). Nakamura et al. demonstrated the importance of autologous APCs in the efficiency of expanding electroporation/piggyBac transposon-generated HER2-specific CAR-T cells (19). Positive effects included an enhanced percentage of CAR-T cells with an early memory phenotype and the avoidance of early T cell exhaustion. Saito et al. described an improved piggyBac-transposons-based protocol for the production of CD19 CAR-T in the presence of autologous feeder cells (20). Additionally, similarly to our report, Ramanayake et al. developed an efficient protocol for selective antigen-specific CAR19 expansion by stimulation with an allogeneic feeder without polyclonal anti-CD3/CD28 stimulation (22). Our protocol, however, uses different combination of cytokines (i.e. IL-4, IL-7, IL-21) plus polyclonal stimulation with TransAct that leads in our experience to expansion of CAR-T with enhanced early-memory phenotype and is more efficient than expansion in the presence of IL-7 and IL-15 (data not shown) (8, 23).

In summary, we described an improved cultivation technique based on the addition of irradiated, mixed allogeneic PBMCs that improves the efficiency of CAR-T manufacturing by transposon/electroporation based methods especially in lymphopenic patients who recently underwent intensive chemotherapies for B-cell lymphomas and acute myeloid leukemia, respectively. The effect of the allogeneic feeder was based mainly on the enhancement of overall T cell expansion after electroporation. The allogeneic mixed feeder cells can be easily produced from healthy blood-donors using the buffy coats and this method is technically feasible and acceptable for the regulatory authorities.

## Data Availability

The raw data supporting the conclusions of this article will be made available by the authors, without undue reservation.
