# Corrigendum: Good manufacturing practice-grade generation of CD19 and CD123-specific CAR-T cells using piggyBac transposon and allogeneic feeder cells in patients diagnosed with B-cell non-Hodgkin lymphoma and acute myeloid leukemia

**DOI:** 10.3389/fimmu.2024.1483043

**Published:** 2024-09-09

**Authors:** Martin Mucha, Martin Štach, Iva Kaštánková, Jana Rychlá, Jan Vydra, Petr Lesný, Pavel Otáhal

**Affiliations:** ^1^ Institute of Hematology and Blood Transfusion, Prague, Czechia; ^2^ Faculty of Science, Charles University, Prague, Czechia

**Keywords:** CAR-T cells, leukemia, lymphoma, electroporation, PiggyBac PB transposon

In the published article, there was an error in **Figure 2** as published. The description under **Figure 2A** was displayed as “PBMC:feeder ratio”.

**Figure 2:**
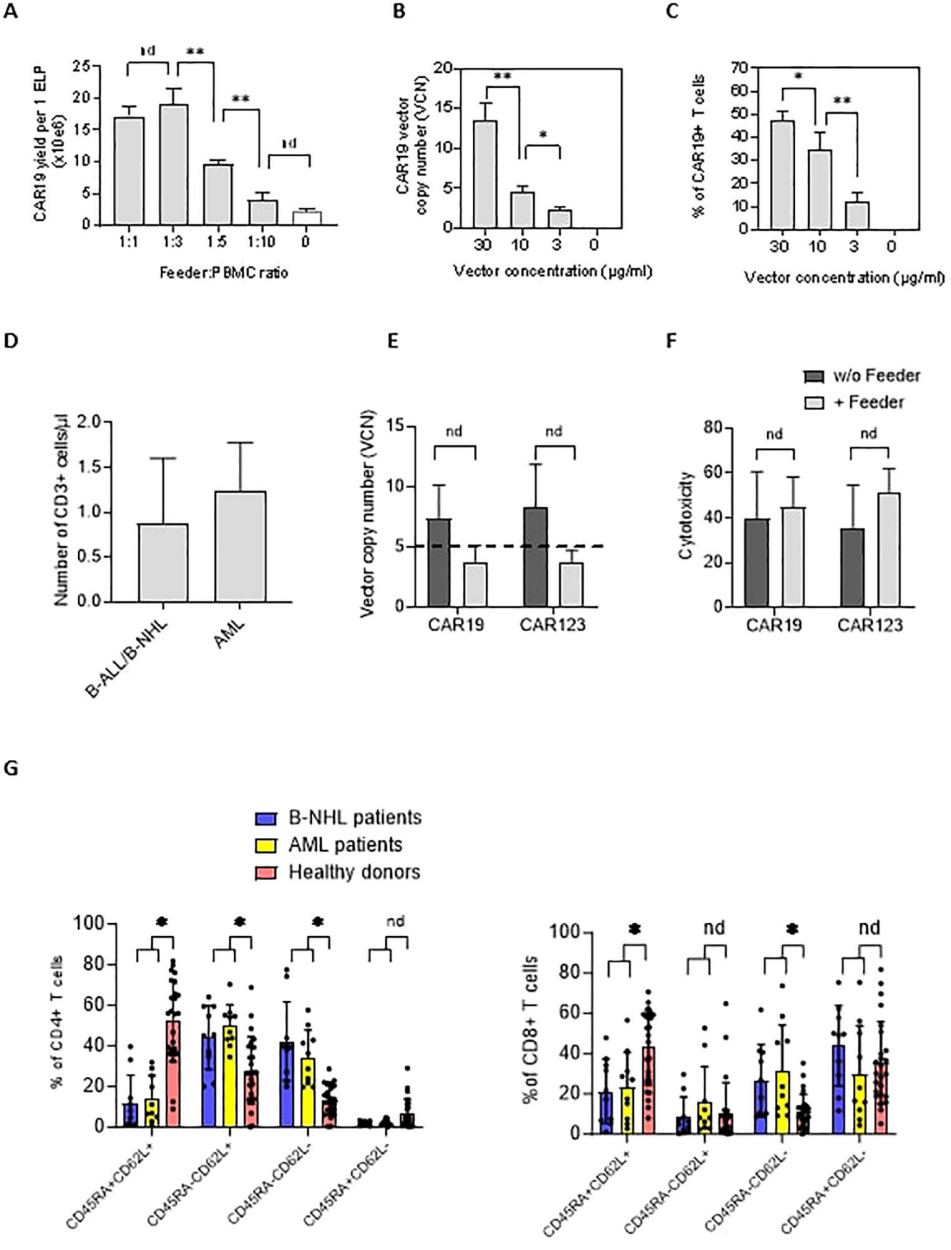
The effects of the feeder on the quality of the produced CAR19 and CAR123 T cells. **(A)** The PBMCs from B-NHL patients (n=3) were electroporated with CAR19 transposon and mixed with decreasing amounts of the feeder. The number of CAR+ T cells was determined after 14 days of expansion as a yield per one ELP. The optimal adequate amount of the feeder improving the CAR-T production was estimated to be at a 1:3 feeder: PBMCs ratio. **(B, C)** The concentration of the transposon DNA during electroporation influences the vector copy number (VCN) and the percentage of transfected T cells. The optimal concentration of the transposon vector to meet the VCN limits=5 and to enable effective transfection was determined to be 10 µg/ml (n=3). **(D)** The graph presents the median number and range of CD3+ T lymphocytes in blood samples used for low-scale production of CAR-T cells obtained from B-NHL and AML patients (n=10). Both groups of patients were lymphopenic as a result of previous chemotherapies. **(E)** To evaluate the effects of the feeder on the transposition efficiency, we measured the vector copy number (VCN) per one CAR19+ and CAR123+ T cell in the presence or absence of the feeder (vector concentration =10 µg/ml, PBMCs were obtained from B-NHL and AML patients (n=4)). The differences in VCN were insignificant due to the high variability of the VCN in CAR-T expanded without the feeder. However, all products expanded in the presence of the feeder had acceptable VCN (≤5). **(F)** The biological activity of produced CAR-T in the presence or absence of the feeder was determined by cytotoxic assay against RAMOS cells (CAR19) or THP-1 cells (CAR123) at 1:1 effector: target ratio after 24 hours of co-culture - no significant differences in the cytotoxicity between feeder/no-feeder produced CAR19, and CAR123 T cells were observed (n=4, nd = no difference, unpaired t test). **(G)** The T cell memory phenotype was determined to evaluate the effects of chemotherapies on the quality of T cells by staining for antigens CD45RA and CD62L on CD4+ or CD8+ T cells. Patient-derived samples contained significantly fewer CD45RA+CD62L+ T cells and significantly more T cells having more differentiated phenotype CD45RA-CD62L- in both CD4+ and CD8+ subsets, reflecting the patients' conditions. B-NHL n=8, AML n=10, **P < 0.01, *P < 0.05, nd = no difference, +/- SD, unpaired t test.

Correct description is “Feeder: PBMC ratio”.

The authors apologize for this error and state that this does not change the scientific conclusions of the article in any way. The original article has been updated.

